# Effects of the *CDC10* (*Septin 7*) Gene on the Proliferation and Differentiation of Bovine Intramuscular Preadipocyte and 3T3-L1 Cells

**DOI:** 10.3390/ani13040609

**Published:** 2023-02-09

**Authors:** Zixuan Cheng, Xihe Li, Siqin Bao, Takahisa Yamada, Guifang Cao, Jianfeng Liu, Aorigele Chen, Bin Tong

**Affiliations:** 1 The State Key Laboratory of Reproductive Regulation and Breeding of Grassland Livestock, School of Life Sciences, Inner Mongolia University, Hohhot 010070, China; 2 Department of Agrobiology, Faculty of Agriculture, Niigata University, Niigata 950-2181, Japan; 3 Department of Veterinary, Inner Mongolia Agricultural University, Hohhot 010018, China; 4 National Engineering Laboratory for Animal Breeding and MOA Key Laboratory of Animal Genetics and Breeding, College of Animal Science and Technology, China Agricultural University, Beijing 100193, China; 5 College of Animal Science, Inner Mongolia Agricultural University, Hohhot 010018, China

**Keywords:** bovine intramuscular preadipocyte, *CDC10*, differentiation, proliferation, *Septin 7*, 3T3-L1

## Abstract

**Simple Summary:**

Intramuscular fat content and marbling affecting meat quality are important economic traits in beef cattle. *CDC10* (cell division cycle 10 or *Septin 7*), a member of the septin family involved in cellular proliferation, was considered as a functional and positional candidate gene for beef marbling. In the present study, we found that overexpression of *CDC10* could promote the differentiation of bovine intramuscular preadipocyte and 3T3-L1 cells, whereas knockdown of *CDC10* resulted in the opposite consequences. Our results provide new insights into the regulatory roles of *CDC10* in adipocytes in animals.

**Abstract:**

Intramuscular fat content and marbling affecting meat quality are important economic traits in beef cattle. *CDC10* (cell division cycle 10 or *Septin 7*), a member of the septin family involved in cellular proliferation, was considered as a functional and positional candidate gene for beef marbling. In a previous study, we revealed that the expression levels of *CDC10* were also positively correlated with marbling scores in Japanese Black cattle. However, the regulatory mechanism of the *CDC10* gene on IMF deposition in cattle remains unclear. In the present study, flow cytometry, EdU proliferation assays, and Oil Red O staining results showed that overexpression of *CDC10* could promote the differentiation of bovine intramuscular preadipocyte (BIMP) and 3T3-L1 cells, whereas knockdown of *CDC10* resulted in the opposite consequences. Furthermore, quantitative PCR and Western blotting results showed that overexpression of *CDC10* could promote the expression levels of adipogenic marker genes *PPARγ* and *C*/*EBPα* at both mRNA and protein levels in BIMP and 3T3-L1 cells, whereas knockdown of *CDC10* resulted in the opposite consequences. Our results provide new insights into the regulatory roles of *CDC10* in adipocytes in animals.

## 1. Introduction

Intramuscular fat (IMF) content and marbling are important economic traits in the beef cattle industry. IMF is the streak of visible fat intermixed with the lean within a muscle fiber of different muscle types and locations [1]. It was found that the sensory quality traits of beef, such as tenderness, juiciness, taste, and flavor, were related to IMF content and marbling [2]. Moreover, IMF content is a complex quantitative trait affected by multiple genetic components, cellular signals, environmental conditions, and hormones [3,4]. To date, a number of studies have demonstrated that many candidate genes and mutations play important roles in regulating the deposition of IMF [5,6,7,8,9,10,11]. However, a better knowledge of the molecular architecture of IMF content and marbling is advantageous as it may provide effective scientific clues for improving meat quality, leading to economic benefits to the beef production industry.

Cell division cycle 10 (CDC10, also known as Septin 7) is a member of the septin family, which belongs to a family of cytoskeleton proteins with GTPase activity assembled as intracellular filamentous scaffolds [12]. To date, 13 different septin genes (SEPT1–SEPT12 and SEPT14) have been identified in mammals [13] and can be classified into four different subgroups (SEPT2, SEPT3, SEPT6, and SEPT7 subgroups) based on the sequence homology of their structural domains [14,15]. They play putative roles in cytokinesis, cellular morphogenesis, polarity determination, vesicle trafficking, and apoptosis [16,17,18,19]. Among them, the *CDC10* gene is essential for cytokinesis in a variety of cells and is involved in cellular proliferation [20,21,22]. As reported, knockdown of the *CDC10* gene results in fewer cells entering S-phase in the cell model and leads to abnormal mitotic spindle formation, but mitotic spindle abnormalities can be rescued by treatment with CDC10 protein; thus, the *CDC10* gene is important for maintaining spindle formation [23]. *CDC10* is related to the phosphorylation of MEK/ERK, which is a key signaling pathway for adipocyte differentiation. MEK/ERK activity is necessary for the expression of key adipogenic regulators CCAAT/enhancer binding proteins α (*C*/*EBPα*) and peroxisome proliferator-activated receptor γ (*PPARγ*) [24,25]. Furthermore, *CDC10* could interact with fat storage-inducing transmembrane protein 2 (FIT2), which is a key gene that packs fat into lipid droplets [26] to induce their formation [27]. Therefore, these studies suggested that the *CDC10* gene may play a critical role in adipogenesis. However, the regulatory role of *CDC10* on the proliferation and differentiation of the preadipocyte remains unclear.

The aims of this study were to: (1) identify the growth curve and differentiation ability of primary cultured bovine intramuscular preadipocyte (BIMP); (2) detect the expression trend of the *CDC10* gene during the proliferation and differentiation of BIMP and 3T3-L1 cells; (3) detect the effects on proliferation and differentiation of BIMP and 3T3-L1 preadipocyte after the knockdown or overexpression of the *CDC10* gene. In this study, we initially investigated the mechanism of the *CDC10* gene effect on the proliferation and differentiation of 3T3-L1 and BIMP to provide scientific clues for the improvement of beef quality.

## 2. Materials and Methods

### 2.1. Ethics Standards 

All experimental procedures were carried out in accordance with the guidelines of the China Council on Animal Care. The protocol was approved on 1 March 2018 by the Institutional Animal Care and Use Ethics Committee of Inner Mongolia University, with the permit number for conducting animal experiments IMU-2018-01.

### 2.2. Cell Culture

BIMP were isolated from the *longissimus dorsi* muscle of three 24-month-old healthy Qinchuan steers. The three steers were randomly selected from the slaughterhouse. The isolated adipose tissue was washed three times in phosphate buffered saline (PBS). Then, the tissues were minced and digested with collagenase type I (Sigma-Aldrich, MO, USA) for 1 h at 37 °C in a shaker, and then passed through 50-, 100-, and 250-mesh sieves successively. The preadipocyte were collected with centrifugation at 1500 rpm for 5 min. Bovine preadipocyte were cultured in growth medium consisting of Dulbecco’s Modified Eagle Medium/Nutrient Mixture F-12 (DMEM/F12, Gibco-BRL, Carlsbad, CA, USA) with 1% penicillin-streptomycin (PS, Gibco-BRL, Carlsbad, CA, USA) and 20% fetal bovine serum (FBS, Gibco-BRL, Carlsbad, CA, USA) at 37 °C with 5% CO_2_. The 3T3-L1 preadipocyte were purchased from Procell (Wuhan, China) and were cultured in growth medium consisting of DMEM/F12 with 10% FBS at 37 °C with 5% CO_2_. For BIMP and 3T3-L1 differentiation, when the confluence of cells reached 90%, they were induced to differentiate using a differentiation cocktail (DMEM/F12 with 10% FBS, 0.5 mmol/L 3-Isobutyl-1-methyxanthine (IBMX), 10 μg/mL insulin, 1 μmol/mL dexamethasone (Sigma-Aldrich, MO, USA)) for 3 d. Cells were then cultured in DMEM/F12 with 10% FBS and 10 μg/mL insulin for 3 d. Finally, cells were cultured in DMEM/F12 with 10% FBS and the culture medium was changed every 3 d until day 15. Cells were incubated at 37 °C with 5% CO_2_. 

### 2.3. Transfection of siRNA and Lentivirus Infection

The bovine and murine small-interfering RNA (siRNA) used in this study were designed by RIBOBIO (RIBOBIO, Guangzhou, China) and contained three *CDC10* gene siRNA sequences (Table 1) and an siRNA negative control (NC). Cells were cultured in 6-well plates to reach about 50% confluency prior to transfection and then siRNA was transfected using the ribio*FECT*^TM^CP (RIBOBIO, Guangzhou, China) transfection reagent according to the manufacturer’s protocol.

For overexpression, we constructed two lentiviral overexpression vectors by introducing the coding region sequence of the bovine *CDC10* gene (GenBank accession: BC148893.1) and of the murine *CDC10* gene (GenBank accession: BC058587.1) available on GenBank into the virus plasmid. Constructions of bovine and murine overexpression vectors were completed by Gemma gene (Shanghai, China). In order to establish the stable cell line, the preadipocyte were inoculated with the lentivirus for 96 h and then stable cell lines were screened by flow cytometry assay.

### 2.4. RNA Isolation and Quantitative Real-Time PCR

Total RNA was extracted using RNeasy Mini Kit (Qiagen, Valencia, CA, USA) and then the reverse transcription kit (Takara, Kusatsu, Japan) was used to synthesize cDNA. Quantitative real-time PCR (RT-PCR) was carried out with the use of an SYBR Green master mix and specific primers (Table 2) on a BioRad system (Bio-Rad, Hercules, CA, USA). The relative mRNA abundance of each gene was analyzed by the 2^−ΔΔCT^ method. 

### 2.5. Western Blot Analysis

Cell samples were lysed using radioimmunoprecipitation assay (RIPA) buffer (Beyotime, Shanghai, China) supplemented with phenylmethylsulfonyl fluoride (PMSF) (Beyotime, Shanghai, China) and total protein was extracted. The total protein samples were separated by electrophoresis in SDS-polyacrylamide gels and then transferred to NC membranes (Pall Corporation, East Hills, NY, USA). After blocking the membrane in 5% skim milk for 2 h, the primary antibody was incubated overnight (4 °C) and the secondary antibody was incubated at room temperature for 1 h. Protein bands were exposed with chemiluminescent reagents (Thermo, Waltham, MA, USA). An anti-α-Tubulin antibody was used as the loading control. The protein expression was quantified using ImageJ (https://imagej.nih.gov/ij/, accessed on 18 June 2021). The following primary antibodies were used: CDC10 (1:1000, Abcam), CyclinD1 (1:500, Proteintech), CyclinB1 (1:500, Proteintech), α-Tubulin (1:1000, Proteintech), *C/EBPα* (1:500, Proteintech), and PPARγ (1:500, Proteintech). The secondary antibodies were anti-rabbit (1:10,000, Proteintech) and anti-mouse antibodies (1:10,000, Proteintech). The targeted proteins were detected using the Tanon-5200 (Tanon, Shanghai, China) instructions of the manufacturer.

### 2.6. EdU and Flow Cytometry Assay

We used the Cell-Light™ EdU Apollo^®^ 488 In Vitro Imaging Kit (RIBOBIO, Guangzhou, China) and configured the mixed solution according to the instructions. The preadipocyte in the normal growth stage were treated with 50 μmol/L EdU medium for 2 h. After the cells were fixed with 4% paraformaldehyde, they were stained with the Apollo reaction solution and then the cell nucleus was stained with Hoechst. A Nikon TE2000 microscope (Nikon, Tokyo, Japan) was used to take pictures and the data were analyzed using ImageJ.

Preadipocyte were seeded in 6-well culture plates at a density of 2 × 10^5^ cells per well. Cells were transfected with *CDC10*-siRNA or a *CDC10* overexpression vector. After washing three times with PBS, cells were fixed with 75% alcohol overnight at 4 °C, followed by treatment with 1 mg/mL RNase at 37 °C for 30 min, and were then stained with 50 mg/mL propidium iodide (PI) at 37 °C for 30 min. The samples were detected using a CytoFLEX flow cytometer (Becton Dickinson, Franklin Lakes, NJ, USA). The proliferation index shows the proportion of mitotic cells among the cells examined.

### 2.7. Oil Red O Staining

The *CDC10*-siRNA- or *CDC10* overexpression vector-treated cells were matured for 9 d and then washed with PBS, fixed with 4% paraformaldehyde (PFA) for 0.5 h at room temperature, and washed again three times with PBS. After staining with Oil Red O (Sigma-Aldrich, MO, USA) solution for 1 h, the results were visualized on a fluorescence microscope (Nikon, Tokyo, Japan). Oil Red O was extracted with 100% isopropanol and its relative concentration was determined by measuring the absorbance at 510 nm.

### 2.8. Statistical Analysis

All charts were created using GraphPad Prism 7.0 and Origin 2019b. The data represent the mean ± SEM. The significance of differences between the groups was assessed using the Student’s *t*-test (*, *p* < 0.05; **, *p* < 0.01).

## 3. Results

### 3.1. Growth Curve of Primary Cultured BIMP and Identification of Adipogenic Marker Genes

**Figure 1:**
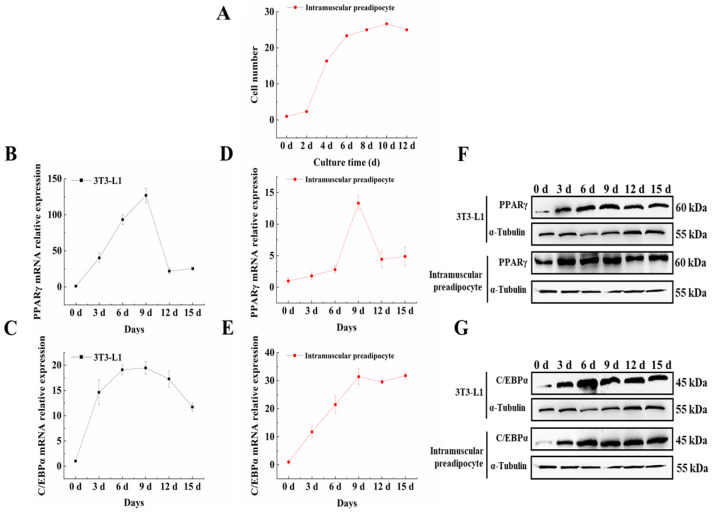
Growth curve of primary cultured bovine intramuscular preadipocyte (BIMP) and identification of adipogenic marker genes. (**A**) Growth curve of BIMP. (**B**–**E**) Expression of *PPARγ* and *C/EBPα* mRNA during the differentiation of BIMP and 3T3-L1 cells. (**F**,**G**) Protein expression of *PPARγ* and *C/EBPα* during the differentiation of BIMP and 3T3-L1 cells. RT-PCR data was normalized to day 0.

In order to identify the growth of primary cultured BIMP and whether they can stably differentiate into adipocytes, we measured the growth curve of BIMP and also detected the expression trends of adipogenic marker genes during their differentiation. The growth curve of BIMP was drawn by trypan blue staining and the results are shown in Figure 1A. The growth curve of BIMP showed an “S” shape (Figure 1A). We found that the expression of *PPARγ* and *C/EBPα* increased first and then decreased during the differentiation in BIMP and 3T3-L1 cells (Figure 1B–E). The levels of PPARγ and *C/EBPα* proteins in the preadipocyte followed a similar trend as the mRNA expression (Figure 1F,G). 

### 3.2. Expression of the CDC10 Gene in the Proliferation and Differentiation of BIMP and 3T3-L1 Cells

**Figure 2:**
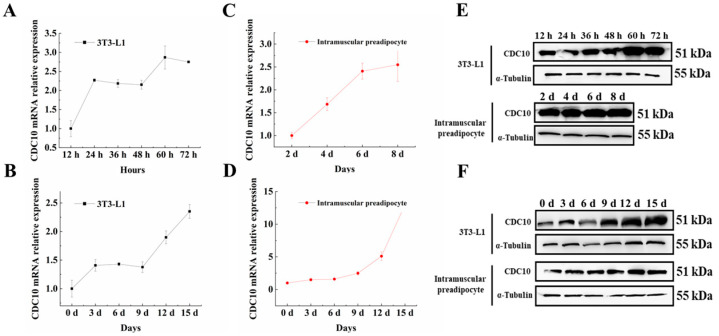
Expression of the *CDC10* gene in the proliferation and differentiation of bovine intramuscular preadipocyte (BIMP) and 3T3-L1 cells. (**A**,**C**) Expression of *CDC10* mRNA during the proliferation of BIMP and 3T3-L1 cells. (**B**,**D**) Expression of *CDC10* mRNA during the differentiation of BIMP and 3T3-L1 cells. (**E**,**F**) Protein expression of CDC10 during the proliferation and differentiation of BIMP and 3T3-L1 cells. RT-PCR data was normalized to day 0, hour 12, and day 2.

In order to unveil the function of *CDC10* in preadipocyte in animals, both 3T3-L1 and BIMP cells were used to analyze *CDC10* expression during preadipocyte proliferation and differentiation. We found that *CDC10* expression increased during proliferation and differentiation in both cell types. Then, *CDC10* mRNA levels peaked at day 8 during BIMP proliferation and peaked at hour 60 during 3T3-L1 proliferation (Figure 2A,C). Meanwhile, *CDC10* mRNA levels continued to rise until day 15 in BIMP and 3T3-L1 differentiation (Figure 2B,D). The levels of CDC10 protein in the preadipocyte followed a similar trend as the mRNA expression (Figure 2E,F).

### 3.3. Knockdown of CDC10 Had No Significant Effect on the Proliferation of BIMP and 3T3-L1 Cells

**Figure 3:**
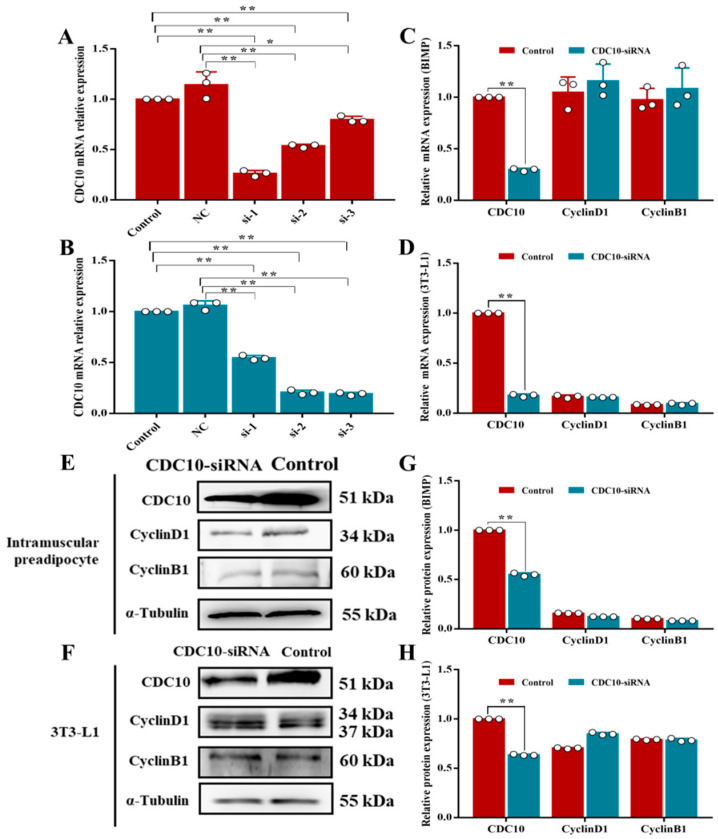
The effect of *CDC10* knockdown on the proliferation of bovine intramuscular preadipocyte (BIMP) and 3T3-L1 cells was detected by RT-PCR and Western blot assay. BIMP and 3T3-L1 cells were transfected with *CDC10*-siRNA for 48 h during proliferation. (**A**) Detection of *CDC10* interference efficiency in BIMP. (**B**) Detection of *CDC10* interference efficiency in 3T3-L1 cells. (**C**,**D**) After interference with *CDC10*-siRNA for 48 h, the expression levels of *CDC10*, CyclinD1, and *CyclinB1* in BIMP and 3T3-L1 cells were determined by RT-PCR and normalized to the *β-actin* level. (**E**,**F**) Protein levels were assessed by Western blot analysis. (**G**,**H**) Quantitative analysis of CDC10, CyclinD1, and CyclinB1 proteins in BIMP and 3T3-L1 cells. Values are expressed as mean ± SEM (*n* = 3). * *p* < 0.05, ** *p* < 0.01. RT-PCR data was normalized to control groups of *CDC10*.

**Figure 4:**
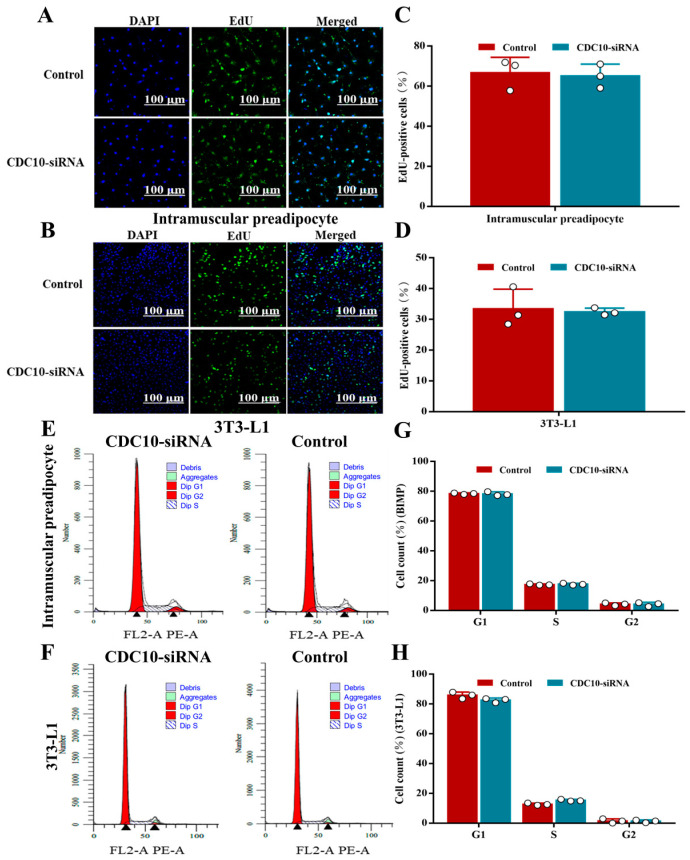
The effect of *CDC10* knockdown on the proliferation of bovine intramuscular preadipocyte (BIMP) and 3T3-L1 cells was detected by EdU and flow cytometry assay. BIMP and 3T3-L1 cells were transfected with *CDC10*-siRNA for 48 h during proliferation. (**A**,**B**) The proliferations of BIMP and 3T3-L1 cells were examined by EdU immunofluorescent staining. Green color represents EdU staining and blue color represents cell nuclei stained with Hoechst 33342. (**C**,**D**) The percentage of EdU-positive cells were quantified. (**E**–**H**) Cell cycle phase analysis of BIMP and 3T3-L1 cells by flow cytometry and their statistical results. Values are expressed as mean ± SEM (*n* = 3).

The interference efficiencies of the six siRNAs were detected by RT-PCR. The results indicated that *CDC10*-siRNA1 in BIMP had the strongest interference efficiency, with 81% and 82% compared to control and NC, respectively (Figure 3A), and *CDC10*-siRNA3 in 3T3-L1 had the strongest interference efficiency, with 74% and 77% compared to control and NC, respectively (Figure 3B). The interference efficiency in both cell types was greater than 70% and could be used for subsequent experiments. Compared with the control group, knockdown of *CDC10* had no significant effect on the expression of CyclinD1 and CyclinB1 in both BIMP and 3T3-L1 cells (Figure 3C,D). Similarly, knockdown of *CDC10* had no significant effect on the protein levels of *CyclinD1* and *CyclinB1* in both BIMP and 3T3-L1 cells (Figure 3E–H). Knockdown of *CDC10* had no significant effect in the percentage of EdU-positive BIMP and 3T3-L1 cells compared with the control groups (Figure 4A–D). Moreover, we found using flow cytometry that the proportion of G1, S, and G2 phase cells was not significantly changed in both BIMP and 3T3-L1 cells following knockdown of *CDC10* (Figure 4E–H).

### 3.4. Overexpression of CDC10 Had No Significant Effect on the Proliferation of BIMP and 3T3-L1 Cells

**Figure 5:**
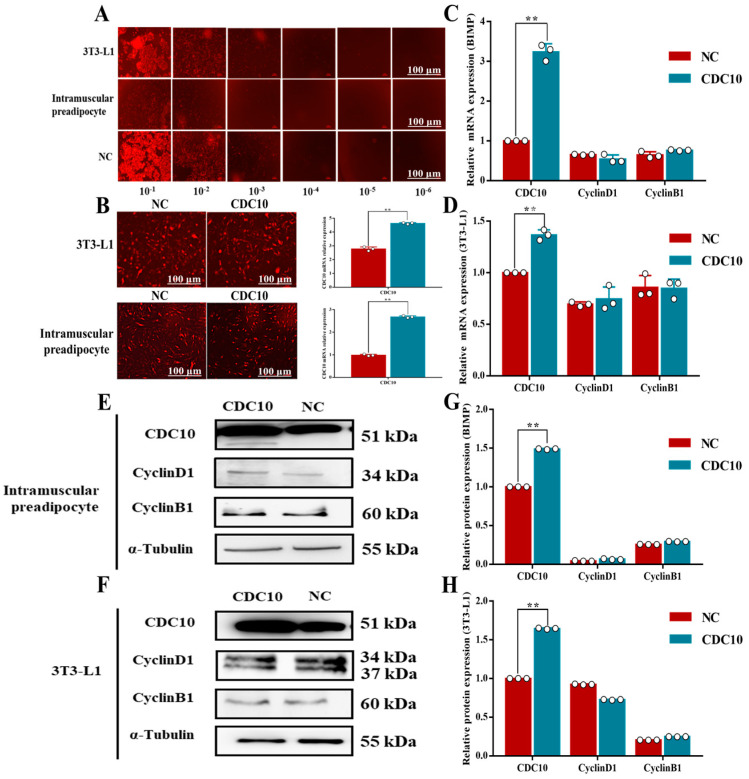
The effect of *CDC10* overexpression on the proliferation of bovine intramuscular preadipocyte (BIMP) and 3T3-L1 cells was detected by RT-PCR and Western blot assay. BIMP and 3T3-L1 cells were transfected with lentivirus for 96 h during proliferation. (**A**) Determination of lentivirus titer. (**B**) Quantitative detection of the overexpression efficiency of *CDC10* in BIMP and 3T3-L1 cells. (**C**,**D**) After overexperssion with lentivirus for 96 h, the expression levels of *CDC10*, *CyclinD1*, and *CyclinB1* in BIMP and 3T3-L1 cells were determined by RT-PCR and normalized to the *β-actin* level. (**E**,**F**) Protein levels were assessed by Western blot analysis (small band of *CDC10* in (**E**) is a non-specific band). (**G**,**H**) Quantitative analysis of CDC10, CyclinD1, and CyclinB1 proteins in BIMP and 3T3-L1 cells. Values are expressed as mean ± SEM (*n* = 3). ** *p* < 0.01. RT-PCR data was normalized to control groups of *CDC10*.

**Figure 6:**
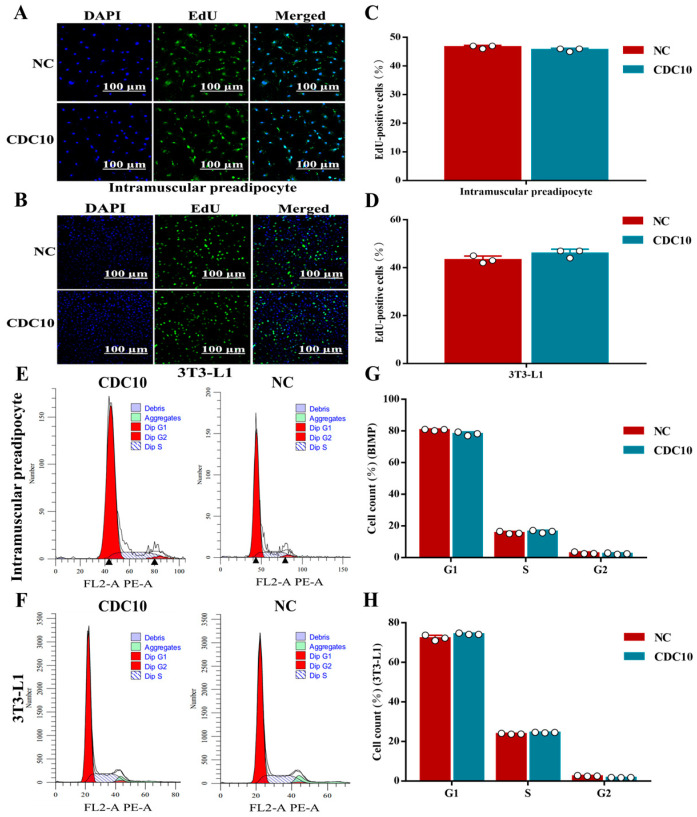
The effect of *CDC10* overexpression on the proliferation of bovine intramuscular preadipocyte (BIMP) and 3T3-L1 cells was detected by EdU and flow cytometry assay. BIMP and 3T3-L1 cells were transfected with lentivirus for 96 h during proliferation. (**A**,**B**) The proliferations of BIMP and 3T3-L1 cells were examined by EdU immunofluorescent staining. Green color represents EdU staining and blue color represents cell nuclei stained with Hoechst 33342. (**C**,**D**) The percentage of EdU-positive cells were quantified. (**E**–**H**) Cell cycle phase analysis of BIMP and 3T3-L1 cells by flow cytometry and their statistical results. Values are expressed as mean ± SEM (*n* = 3).

The titer of overexpressed virus was detected by gradient dilution method. The results show that lentivirus titers of BIMP, 3T3-L1, and NC were 1.1 × 10^−6^, 3.1 × 10^−6^, and 1.6 × 10^−7^, respectively. Therefore, these lentiviruses could be used in subsequent tests (Figure 5A). BIMP and 3T3-L1 cells were infected with lentivirus for 96 h. GFP fluorescence was found throughout the field of view and the results showed that BIMPs were effectively transfected (Figure 5B). Compared with the control group, overexpression of *CDC10* had no significant effect on the expression of *CyclinD1* and *CyclinB1* in BIMP and 3T3-L1 cells (Figure 5C,D). Similarly, overexpression of *CDC10* had no significant effect on the protein levels of *CyclinD1* and *CyclinB1* in BIMP and 3T3-L1 cells (Figure 5E–H). Stable cells were screened by flow cytometry after infection. Overexpression of *CDC10* had no significant effect in the percentage of EdU-positive BIMP and 3T3-L1 cells compared with the control group (Figure 6A–D). Moreover, we found using flow cytometry that the proportion of G1, S, and G2 phase cells were not significantly changed in BIMP and 3T3-L1 cells following overexpression of *CDC10* (Figure 6E–H).

### 3.5. Knockdown of CDC10 Inhibits Differentiation of BIMP and 3T3-L1 Cells

**Figure 7:**
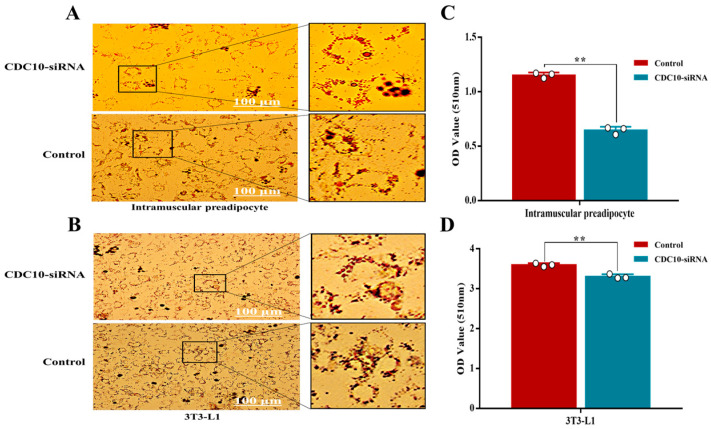
The effect of *CDC10* knockdown on the differentiation of bovine intramuscular preadipocyte (BIMP) and 3T3-L1 cells was detected by Oil Red O staining. BIMP and 3T3-L1 cells were transfected with *CDC10*-siRNA for 9 d during differentiation. (**A**,**B**) Oil Red O staining. (**C**,**D**) Oil Red O relative absorbance. Values are expressed as mean ± SEM (*n* = 3). ** *p* < 0.01.

**Figure 8:**
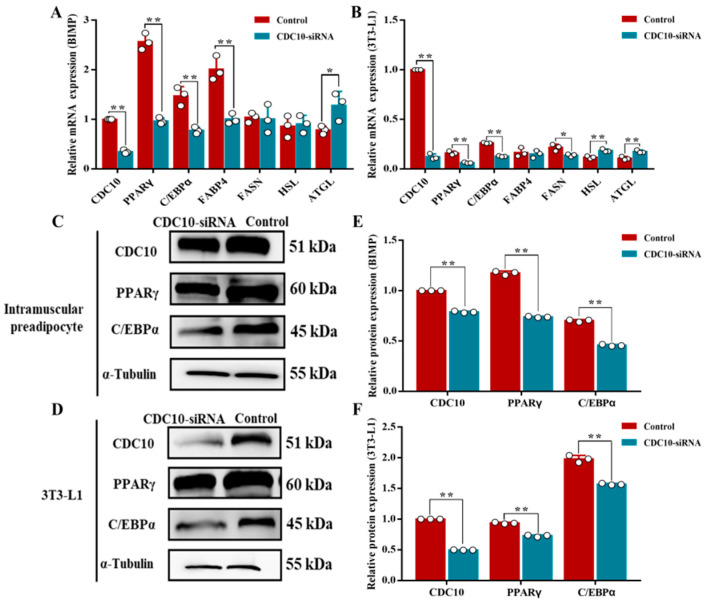
The effect of *CDC10* knockdown on the differentiation of bovine intramuscular preadipocyte (BIMP) and 3T3-L1 cells was detected by RT-PCR and Western blot assay. BIMP and 3T3-L1 cells were transfected with *CDC10*-siRNA for 9 d during differentiation. (**A**,**B**) After interference with *CDC10*-siRNA for 9 d, the expression levels of *CDC10*, *PPARγ*, *C*/*EBPα*, *FABP4*, *FASN*, *HSL*, and *ATGL* were determined by RT-PCR and normalized to the *β-actin* level. (**C**,**D**) Protein levels were assessed by Western blot analysis. (**E**,**F**) Quantitative analysis of CDC10, PPARγ, and *C*/*EBPα* proteins in BIMP and 3T3-L1 cells. Values are expressed as mean ± SEM (*n* = 3). * *p* < 0.05, ** *p* < 0.01. RT-PCR data was normalized to control groups of *CDC10*.

Oil Red O staining revealed that knockdown of *CDC10* led to significantly reduced lipid droplets produced by BIMP and 3T3-L1 cells compared with the control groups (Figure 7A,B). The results of Oil Red O extraction showed that knockdown of *CDC10* led to decreases in the intracellular lipid content of BIMP and 3T3-L1 cells compared with the control groups (*p* < 0.01) (Figure 7C,D). RT-PCR results showed that the expression of *CDC10* was significantly lower than that of the control groups in BIMP and 3T3-L1 cells (*p* < 0.01) (Figure 8A,B). The levels of adipogenic marker genes *PPARγ*, C/EBPα, and fatty acid-binding protein (*FABP4*) were significantly reduced in BIMPs at the mRNA level following knockdown of *CDC10* (*p* < 0.01, *p* < 0.01, and *p* < 0.01, respectively) (Figure 8A), while *PPARγ*, *C/EBPα*, and fatty acid synthetase (*FASN*) were significantly reduced in 3T3-L1 at the mRNA level following knockdown of *CDC10* (*p* < 0.01, *p* < 0.01, and *p* < 0.05, respectively) (Figure 8B). However, expression of adipose triglyceride lipase (*ATGL*) was increased in BIMP (*p* < 0.05) and 3T3-L1 (*p* < 0.01) after knockdown of *CDC10* (Figure 8A,B). Similarly, the protein levels of PPARγ and *C/EBPα* were significantly reduced in BIMP and 3T3-L1 cells after knockdown of *CDC10* (Figure 8C–F).

### 3.6. Overexpression of CDC10 Promotes Differentiation of BIMP and 3T3-L1 Cells

**Figure 9:**
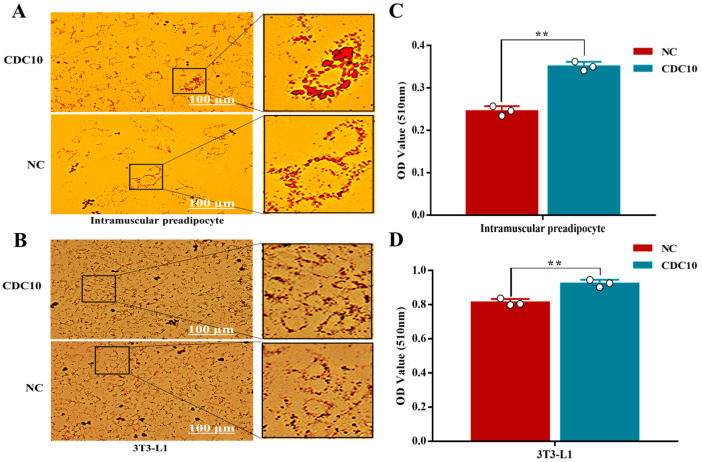
The effect of *CDC10* overexpression on the differentiation of bovine intramuscular preadipocyte (BIMP) and 3T3-L1 cells was detected by Oil Red O staining. BIMP and 3T3-L1 cells were transfected with lentivirus for 9 d during differentiation. (**A**,**B**) Oil Red O staining. (**C**,**D**) Oil Red O relative absorbance. Values are expressed as mean ± SEM (*n* = 3). ** *p* < 0.01.

**Figure 10:**
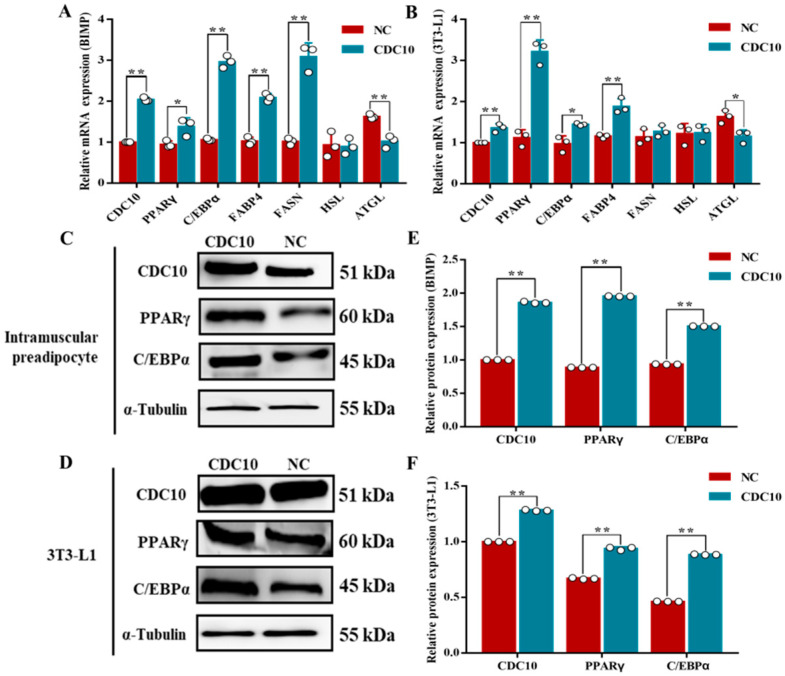
The effect of *CDC10* overexpression on the differentiation of bovine intramuscular preadipocyte (BIMP) and 3T3-L1 cells was detected by RT-PCR and Western blot assay. BIMP and 3T3-L1 cells were transfected with lentivirus for 9 d during differentiation. (**A**,**B**) After overexpression with lentivirus for 9 d, the expression levels of *CDC10*, *PPARγ*, *C*/*EBPα*, *FABP4*, *FASN*, *HSL*, and *ATGL* were determined by RT-PCR and normalized to the *β-actin* level. (**C**,**D**) Protein levels were assessed by Western blot analysis. (**E**,**F**) Quantitative analysis of *CDC10*, PPARγ, and *C/EBPα* proteins in BIMP and 3T3-L1 cells. Values are expressed as mean ± SEM (*n* = 3). * *p* < 0.05, ** *p* < 0.01. RT-PCR data was normalized to control groups of *CDC10*.

Oil Red O staining revealed that overexpression of *CDC10* led to significantly increased lipid droplets produced by BIMP and 3T3-L1 cells compared with the control groups (Figure 9A,B). The results of Oil Red O extraction showed that overexpression of *CDC10* led to increases in the intracellular lipid content of BIMP and 3T3-L1 cells compared with the control group (*p* < 0.01) (Figure 9C,D). RT-PCR results showed that the expression of *CDC10* was significantly higher than that of the control group (*p* < 0.01) (Figure 10A,B). The levels of adipogenic marker genes *PPARγ*, *C/EBPα*, *FABP4*, and *FASN* were significantly increased in BIMPs at the mRNA level following overexpression of *CDC10* (*p* < 0.05, *p* <0.01, *p* < 0.01, and *p* < 0.01, respectively) (Figure 10A), while *PPARγ*, *C/EBPα*, and *FABP4* were significantly increased in 3T3-L1 at the mRNA level following overexpression of *CDC10* (*p* < 0.01, *p* < 0.05, and *p* < 0.01, respectively) (Figure 10B). However, expression of *ATGL* was decreased in BIMP (*p* < 0.01) and 3T3-L1 cells (*p* < 0.05) after overexpression of *CDC10* (Figure 10A,B). Similarly, the protein levels of PPARγ and *C/EBPα* were significantly increased in BIMP and 3T3-L1 cells after overexpression of *CDC10* (Figure 10C–F).

## 4. Discussion

Marbling is an important determinant of beef quality and nutrient value. Therefore, it is essential to understand the mechanism of IMF deposition in beef cattle [4,28]. Intramuscular fat deposition involves a series of events that initiate and maintain preadipocyte proliferation, differentiation of preadipocyte into adipocytes, and adipocyte maturation in muscle [4,29]. In this study, we first used 3T3-L1 and BIMP cells as experimental materials, interfered with the *CDC10* gene expression by siRNA, overexpressed the *CDC10* gene by constructing a *CDC10* gene lentiviral overexpression vector, and detected the effects on proliferation and differentiation of 3T3-L1 and BIMP cells after interfering and overexpressing the *CDC10* gene. Eventually, we first demonstrated that *CDC10* could promote the differentiation of BIMP and 3T3-L1 cells, providing new clues to reveal the mechanism of intramuscular fat formation. 

### 4.1. Effect of the CDC10 Gene on Proliferation of BIMP and 3T3-L1 Cells

CDC10 could be considered as a centrosome protein that plays an important role in maintaining cell proliferation [23], and knockdown of the *CDC10* gene significantly reduced the proliferation rate of breast cancer cells [22]. Studies on C2C12 cells found that the *CDC10* gene is the direct target of miR-127-3p and overexpression of miR-127-3p could significantly inhibit the proliferation of C2C12 cells, whereas interference with miR-127-3p could significantly promote the proliferation of C2C12 cells by regulating the expression of *CDC10* [30]. However, there was no effect of *CDC10* on the proliferation of 3T3-L1 and BIMP cells by the EdU proliferation assay and the cell cycle-related assay, and also no influence on the expressions of *CyclinD1* and *CyclinB1* in this study. Thus, we speculate that *CDC10* may differentially regulate proliferation in different cell types.

### 4.2. Effect of the CDC10 Gene on the Differentiation of BIMP and 3T3-L1 Cells

Adipocyte differentiation refers to the accumulation of fat and morphological changes achieved through the expression of a series of lipogenesis-related genes under the regulation of multiple transcription factors. CDC10 can form a reciprocal relationship by binding to the *FIT2* gene, which is a key gene for packing fat into lipid droplets and is highly expressed in adipose tissue. During adipocyte differentiation, *CDC10* and *FIT2* are transiently enriched in lipid droplet formation sites, and the *CDC10* gene can influence the formation of new lipid droplets by promoting the expression of *FIT2* [27]. This is consistent with our findings that *CDC10* can promote the differentiation of 3T3-L1 cells and bovine adipocytes that we observed through the Oil Red O stain assay, which showed that overexpression of *CDC10* could increase the formation of lipid droplets, whereas knockdown of *CDC10* could decrease formation of lipid droplets in BIMP and 3T3-L1 cells. 

In addition, the expression levels of adipogenesis-related transcription factors and their activity could crucially determine the differentiation process of adipocytes. Among them, *PPARγ* plays an important role in the regulation of adipocyte differentiation, glucolipid metabolism, and insulin signaling. As reported, *PPARγ* could directly affect a number of genes related to lipid storage in adipocytes and is necessary for adipocyte differentiation [31]. *C/EBPα* is induced in the late stage of adipogenesis and is most abundant in mature adipocytes [32]. *C/EBPα* could initiate transcription of adipocyte-specific genes and promotes adipocytes to the terminal differentiation stage [33]. In this study, we first found that the expression levels of *CDC10* had an upward trend during the differentiation of BIMP and 3T3-L1 cells. We also found that overexpression of *CDC10* could increase the expression levels of *PPARγ* and *C/EBPα*, whereas knockdown of *CDC10* could decrease the expression levels of *PPARγ* and *C/EBPα* in BIMP and 3T3-L1 cells, suggesting that the *CDC10* gene has an effect on adipogenesis. Meanwhile, *ATGL* is the key rate-limiting enzyme involved in the breakdown of fat cells [34]. The main function of *ATGL* in adipocytes is to regulate lipid droplet size and fat degradation in adipocytes by catalyzing the hydrolysis of triglycerides in adipocytes [35,36]. In this study, overexpression of *CDC10* could decrease the expression of *ATGL*, whereas knockdown of *CDC10* could increase the expression of *ATGL* in BIMP and 3T3-L1 cells. Since *PPARγ* and *C/EBPα* are key genes involved in adipogenesis and *ATGL* is a key gene involved in adipocyte catabolism, we speculate that the promotion of 3T3-L1 and BIMP cell differentiation by *CDC10* is achieved by promoting the expressions of the *PPARγ* and *C/EBPα* genes and suppressing the expression of the *ATGL* gene.

Besides, *CDC10* depletion could specifically impair ERK1/2 phosphorylation and *CDC10* is required for MEK/ERK activation [22]. The ERK1/2 pathway activation could accelerate preadipocyte proliferation and promote their differentiation, and ERK1/2 is required for *PPARγ* and *C/EBPα* gene transcription during adipocyte differentiation [37]. Similarly, the expression levels of *CDC10* were also positively correlated with marbling scores in Japanese Black cattle [38]. In summary, we speculate that the *CDC10* gene could regulate adipogenesis through promoting the expression of *PPARγ* and *C/EBPα* by regulating the MEK/ERK signaling pathway in IMF of marbling in beef cattle. 

## 5. Conclusions

In conclusion, this is the first study to demonstrate the regulatory mechanism of the *CDC10* gene in the process of preadipocyte proliferation and differentiation. We found that overexpression of *CDC10* could promote the differentiation of BIMP and 3T3-L1 cells, whereas knockdown of *CDC10* resulted in the opposite consequences. These findings provide new opportunities for improving the meat quality of beef by regulating intramuscular fat deposition and help us to better understand the role and regulatory mechanism of the *CDC10* gene in IMF adipogenesis.

## Figures and Tables

**Table 1:** Bovine and murine *CDC10* siRNA target sequences.

Name	Target Sequence (5′-3′)
si-*B*-*CDC10*-01	CAGAGGAATGCCAACAGTT
si-*B*-*CDC10*-02	CCTGATAACAGAGTGCAAT
si-*B*-*CDC10*-03	GCTGTGGTAGGTAGTAATA
si-*M*-*CDC10*-01	GCTTTGCCAACCTCCCAAA
si-*M*-*CDC10*-02	CCAGAGTATCCAGGACCTT
si-*M*-*CDC10*-03	GGACATGGACTTAAACCAT

*B*: *Bos taurus*; *M*: *Mus musculus*.

**Table 2:** Primers for real-time PCR.

Name	Primer Sequence-F (5′-3′)	Primer Sequence-R (5′-3′)
*B-CDC10*	TCTGAAGCTGAGCTCCAA	AAATTGGTCAAACGGATCCA
*B-CyclinD1*	CTGGTCCTGGTGAACAAACT	ACAGAGGGCAACGAAGGT
*B-CyclinB1*	GGATACCTATGTGCCCAAGAAG	GACGGCGACCCAGACTAAA
*B-PPARγ*	TGGAGACCGCCCAGGTTTGC	AGCTGGGAGGACTCGGGGTG
*B-C*/*EBPα*	TGCGCAAGAGCCGGGACAAG	ACCAGGGAGCTCTCGGGCAG
*B-FABP4*	TCCTTCAAATTGGGCCAGGAA	CCCTTGGCTTATGCTCTCTCA
*B-HSL*	GAGTTTGAGCGGATCATTCA	TGAGGCCATGTTTGCTAGAG
*B-ATGL*	TCTGCCTGCTGATTGCTATG	GGCCTGGATAAGCTCCTCTT
*B-FASN*	TAAGGTTCAAATTGCTGCGT	TCCAGAGCGAAGGAGAGATT
*B-β-actin*	ACCACACCTTCTACAACGAG	GAACATGATCTGGGTCATCTTC
*M-CDC10*	CTTACACCAGAGGAATGCCAA	TTTTCAACTTCAGCCACACC
*M-CyclinD1*	ATGTTCGTGGCCTCTAAGATGAAG	CAGAAGCAGTTCCATTTGCAGCAG
*M-CyclinB1*	AAGGTGCCTGTGTGTGAACC	GTCAGCCCCATCATCTGCG
*M-PPARγ*	TTCGCTGATGCACTGCCTAT	GGAATGCGAGTGGTCTTCCA
*M-CEBPα*	GTGTGCACGTCTATGCTAAACCA	GCCGTTAGTGAAGAGTCTCAGTTTG
*M-FABP4*	TGAAATCACCGCAGACGACA	ACACATTCCACCACCAGCTT
*M-HSL*	TGCCCAGGATTGGATGGTTT	GTGAGAACGCTGAGGCTTTG
*M-ATGL*	GGAGGAATGGCCTACTGAACC	ATCCTCTTCCTGGGGGACAA
*M-FASN*	TTGGCCTACACCCAGAGCTA	TTGTGGTAGAAGGACACGGC
*M-β-actin*	GGCTGTATTCCCCTCCATCG	CCAGTTGGTAACAATGCCATGT

*B*: *Bos taurus*; *M*: *Mus musculu*.

## Data Availability

The original contributions presented in the study are included in this article; further inquiries can be directed to the corresponding author.
